# Intelligent Workflow and Software for Non-Target Analysis of Complex Samples Using a Mixture of Toxic Transformation Products of Unsymmetrical Dimethylhydrazine as an Example

**DOI:** 10.3390/molecules28083409

**Published:** 2023-04-12

**Authors:** Anastasia Yu. Sholokhova, Dmitriy D. Matyushin, Oksana I. Grinevich, Svetlana A. Borovikova, Aleksey K. Buryak

**Affiliations:** A.N. Frumkin Institute of Physical Chemistry and Electrochemistry, Russian Academy of Sciences, 31 Leninsky Prospect, GSP-1, 119071 Moscow, Russia

**Keywords:** unsymmetrical dimethylhydrazine, machine learning, retention index, gas chromatography, mass spectrometry, non-target analysis, toxicity

## Abstract

Unsymmetrical dimethylhydrazine (UDMH) is a widely used rocket propellant. Entering the environment or being stored in uncontrolled conditions, UDMH easily forms an enormous variety (at least many dozens) of transformation products. Environmental pollution by UDMH and its transformation products is a major problem in many countries and across the Arctic region. Unfortunately, previous works often use only electron ionization mass spectrometry with a library search, or they consider only the molecular formula to propose the structures of new products. This is quite an unreliable approach. It was demonstrated that a newly proposed artificial intelligence-based workflow allows for the proposal of structures of UDMH transformation products with a greater degree of certainty. The presented free and open-source software with a convenient graphical user interface facilitates the non-target analysis of industrial samples. It has bundled machine learning models for the prediction of retention indices and mass spectra. A critical analysis of whether a combination of several methods of chromatography and mass spectrometry allows us to elucidate the structure of an unknown UDMH transformation product was provided. It was demonstrated that the use of gas chromatographic retention indices for two stationary phases (polar and non-polar) allows for the rejection of false candidates in many cases when only one retention index is not enough. The structures of five previously unknown UDMH transformation products were proposed, and four previously proposed structures were refined.

## 1. Introduction

Unsymmetrical dimethylhydrazine (UDMH, NH_2_N(CH_3_)_2_) is a chemical compound that has been used as an organic rocket propellant in orbital launch vehicles, spacecraft, and ballistic missiles for decades by many countries and space agencies [1]. It also has other applications [2]: in organic synthesis, as an additive to other compounds, and as a basic chemical. The widespread use in the space industry of the fuel pair UDMH–nitrogen dioxide is associated with a high specific impulse, self-ignition upon contact of the fuel components, and also with the fact that the propellant and oxidizer, in this case, are liquid at room temperature and can be stored for a long time.

UDMH itself is a toxic, hostile, and carcinogenic pollutant [3]. The environmental impact and health effects of exposure to this compound were reviewed in multiple previous works [1,3,4]. UDMH was previously introduced into the environment due to the fall of the spent stages of launch vehicles [3]. The stages contain an unused propellant residue, and each launch of such a rocket is associated with environmental pollution [3,4]. In addition, major amounts of UDMH are released due to launch failures [1] and various other accidents in the space industry. The utilization of missiles filled with UDMH is another possible source of contamination. All these factors make UDMH contamination an important environmental issue that needs to be studied.

UDMH was used as a rocket propellant (pure or mixed with hydrazine) in launch vehicles by all major space agencies until the middle of the 2000s, and it is still used in large launch vehicles and missiles by several countries [4]. Spent rocket stages fall both on the ground and in the oceans, and large territories across the world are contaminated [1]. The Arctic region is heavily affected by this contamination [3].

However, the toxicity and danger of UDMH itself are just the tip of the iceberg of problems with this compound. UDMH easily transforms at ambient conditions in contact with water and oxygen, and forms multiple other compounds: many non-aromatic compounds [5,6,7,8,9] and also various five-membered and six-membered nitrogen-containing aromatic heterocyclic compounds [10,11,12,13]. The most well-known UDMH transformation products include *N*-nitrosodimethylamine and 1-methyl-1*H*-1,2,4-triazole [1,6,10]. Every year, more and more new UDMH transformation products are discovered. Several such previous works are presented in Table 1. The transformation products are found in soil and in other environmental samples [1,8,9,10,11,12,13], are produced artificially (to model environment transformation under laboratory conditions) [13,14,15], or are found in other real samples [16,17,18]. UDMH easily forms mixtures of many dozens of transformation products [11,12,13,14,17]. UDMH transformation products have a long [7] environmental half-life and they often have the same or even higher toxicity compared with UDMH itself [7,17]. This enormously diverse unexplored variety of organic pollutants can hide still underestimated environmental danger [17].

The structure elucidation of new UDMH transformation products is a very complicated task. Standard samples are not available in most cases; usually, the discovered products are absent even in the largest chemical databases [16,17]. Preparative isolation with further investigation using nuclear magnetic resonance (NMR) is the most reliable method but it is too laborious for complex mixtures [10,16]. Gas chromatography–mass spectrometry (GC-MS) and high-performance liquid chromatography–mass spectrometry (HPLC-MS) are the most common methods [8,9,10,11,12,13,14,15,16,17]. However, these methods are not able to determine the structure unambiguously (without standard samples) [17], even if high-resolution mass spectrometry (HRMS) is used. The majority of previous works use unreliable approaches to propose the structures of new UDMH transformation products: library search using the NIST GC-MS library [8,11,12,15,18] or structure proposition using only the molecular formula determined with HRMS [13,14] (see Table 1). A library search gives wrong results easily [17], especially taking into account the fact that many transformation products are absent in the databases. The molecular formula alone is not enough; still, there are many [16,17] isomeric UDMH transformation products.

Many works are also devoted to the target analysis of UDMH transformation products (such as *N*-nitrosodimethylamine) in real samples [9,19,20,21] and in artificial mixtures produced in laboratories. In these works, the standard samples of analyzed compounds are usually used. The use of standard samples allows for the provision of almost absolutely reliable qualitative analysis. At the same time, all of these works are limited to several most well-known UDMH transformation products (mostly aliphatic) formed under highly oxidative conditions [2,5,6,22,23,24,25]. The use of standard samples leaves the variety of aromatic UDMH transformation products unexplored. Three of such works [5,6,9] are listed in Table 1. The degradation of UDMH under oxidizing conditions is extensively studied [26,27,28].

Recently, a more reliable workflow was proposed by our team [17]: the combination of HPLC-HRMS and GC-MS with the methods of artificial intelligence was used to crosscheck the proposed structures of new UDMH transformation products. The proposed structure is considered plausible if it meets several criteria: molecular weight (MW) and exact mass match mass spectrometry (MS) data (using soft ionization methods), the fragmentation in MS with an electron ionization (EI) ion source matches a reference or predicted using machine learning (ML) mass spectrum, and the gas chromatographic retention index (RI) matches the predicted using ML or reference RI. Since reference data are unavailable for the vast majority of candidates, the ML models are key elements of the workflow. However, it is still not possible to be sure of the predicted structures. Additional criteria are required. The GC-MS-based approaches can be used only for volatile UDMH transformation products. However, GC-MS is extensively used for this task (see Table 1).

The above-mentioned works [16,17] consider the mixture of UDMH transformation products formed from long-term storage under uncontrolled conditions of wash water formed during the utilization of rockets [16,29]. The wash water is stored in contact with air for a long time before utilization [16,29]. This mixture is an excellent model sample for developing the methodology of UDMH transformation products discovery [17], and generally speaking, for developing the methodology of a non-target analysis of complex mixtures consisting of unknown organic molecules. The mixture contains dozens of compounds of unknown structure [16,17,29], and this study is also devoted to the investigation of this mixture.

The aims of this study are: (i) the critical re-evaluation of the above-mentioned workflow using additional criteria (RI for polar stationary phase and high-resolution MS^2^ mass spectra) for the crosschecking of the proposed structures; (ii) the development of free, open-source, and ready-to-use software for non-target analysis with a graphical user interface (GUI) for the prediction of RI and mass spectra “in one click”, and comparison with the observed data; and (iii) the discovery of new UDMH transformation products and an estimation of their toxicity in silico.

## 2. Results

### 2.1. Workflow for Non-Target Analysis

The workflow used in this work is based on the workflow described in our previous work [17]. In that work, each structure is checked against the following criteria and is considered plausible only if all five criteria are satisfied: (i) the RI on the non-polar stationary phase deviates from the RI predicted using ML or a reference RI of not more than by 70 units; (ii) the electron ionization mass spectrum satisfactorily matches the spectrum predicted using ML or the reference mass spectrum; (iii) the GC-MS mass chromatogram registered using chemical ionization has a chromatographic peak that has a retention time matching the peak observed with EI, and the spectrum matches the molecular weight of a candidate; (iv) there is a chromatographic peak on the HRMS-HPLC mass chromatogram that matches the molecular formula of the considered candidate; and (v) the candidate shares structural peculiarities with other known UDMH transformation products described in the literature as a UDMH transformation product. In this work, two new criteria were added: (vi) the RI on the polar stationary phase deviates from the RI predicted using ML or a reference RI of not more than by 100 units; and (vii) the MS^2^ mass spectrum (tandem mass spectrum) acquired with HRMS does not contradict the proposed structure.

In criterion (vi), a larger threshold value is used compared with criterion (i): 100 units instead of 70. This is related to the lower accuracy of the RI prediction for the polar stationary phases, compared with the prediction for the non-polar stationary phases [30]. The previously published model [30] was used for prediction. This criterion involves the analysis of the mass chromatogram obtained using the polar stationary phase, i.e., each compound that matches all seven criteria was observed on both stationary phases, and the observed spectra on different stationary phases match each other. Due to the different selectivities of the stationary phases, the reliability is additionally increased. In addition, multiple compounds have similar RIs on the non-polar stationary phase and quite different ones on the polar one, or on the contrary, they have similar RIs on the polar stationary phase and different ones on the non-polar one. Such examples are considered in the following sections and prove that this criterion makes sense, and that the use of two stationary phases allows for the achievement of a better plausibility compared with only one stationary phase.

Criterion (vii) is quite essential because the considered electrospray ionization MS^2^ mass spectra have a high resolution, while the electron ionization one-dimensional mass spectra have a low resolution. Such data allow us to propose the structure of the unknown with a high degree of plausibility [31,32], and this approach is widely used in metabolomics [32]. However, the practical use of these data is complicated by several factors. The MS^2^ mass spectra of many UDMH transformation products contain very few peaks, and the available ML software often predicts mass spectra incorrectly. This is related to the following fact: the CFM-ID [33,34,35] software, which is the best one for MS^2^ mass spectra prediction, was trained using a database of metabolites. The typical UDMH transformation products are molecules with a high N/C ratio (e.g., triazoles) and they significantly differ from the metabolites from the CFM-ID training set. This fact causes a low prediction accuracy.

**Figure 1:**
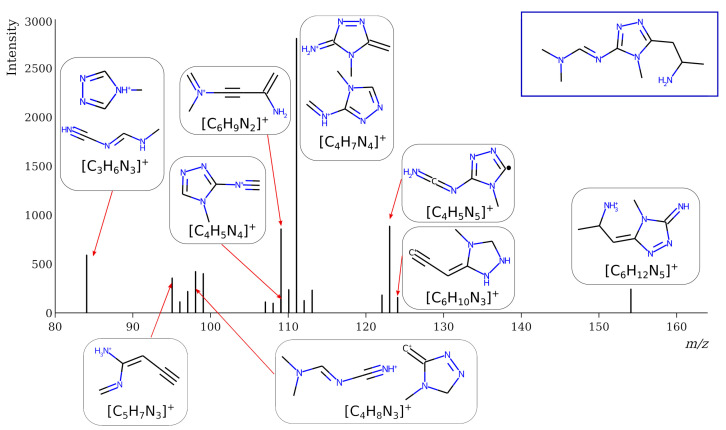
MS^2^ (tandem) mass spectrum and the proposed structure of a newly identified unsymmetrical dimethylhydrazine transformation product. Intensity is given in counts (instrument-dependent arbitrary units); *m*/*z* means mass-to-charge ratio.

However, this criterion still allows for the rejection of false candidates in many cases, e.g., when the observed fragment ion has a molecular formula that cannot be formed from the candidate structure in any reasonable way. In Figure 1, the example of the MS^2^ spectrum of a newly identified UDMH transformation product is shown. The figure also contains structures of fragment ions proposed by the CFM-ID software. This spectrum is consistent with the proposed structure and with CFM predictions.

**Figure 2:**
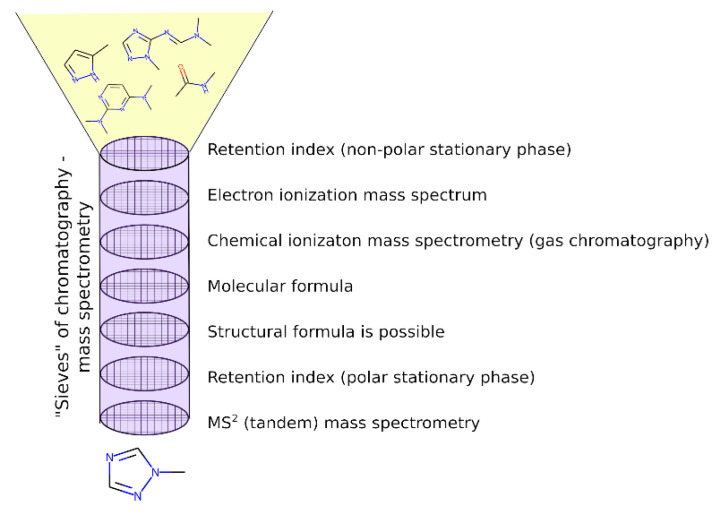
Overview of the workflow for non-target analysis, and the criteria used. Molecular formula and tandem mass spectrometry data were acquired with high performance liquid chromatography–high-resolution mass spectrometry with electrospray ionization.

In this work, compared with the previous work [17], more attention was paid to the UDMH transformation products that are absent in mass spectral databases. In this case, the EI mass spectra predicted with CFM software [33] are used instead of the reference ones presented in the NIST database. The authors of the CFM software used the diverse NIST mass spectral library for training; however, the spectra are predicted incorrectly in many cases. For example, CFM often predicts a high probability of the decay of the bond linking the CH_3_ group, while in practice, the corresponding peak was not observed or was observed at a much lower intensity. Another example is an incorrect prediction of the probability of hydrogen losses. Taking into account these problems with the predicted spectra, we cannot rely only on matching factors (spectral similarity measures between the predicted and the observed spectra). Instead, the observed spectra were analyzed manually and were compared with the predicted ones, and maximal attention was paid to cases when peaks that cannot be produced from the considered structure in any way were observed. The presence of such peaks is a sign that the candidate is false, even if the spectral similarity is high. Therefore, the predicted spectrum is a good starting point to select the candidate structures, but these candidates should be checked manually. Candidate structures, as previously, were extracted according to the determined molecular formulas from the existing chemical and spectral databases, or they were drawn via analogy with known UDMH transformation products. The updated workflow allowed us to critically review the previously proposed structures and to propose more new structures of the UDMH transformation products. The overview of the workflow is depicted in Figure 2. Detailed examples of the application of this workflow are given in the next sections.

### 2.2. Easy-to-Use Software for Non-Target Analysis

The structures of most of the UDMH transformation products constituting the mixture under investigation are absolutely unknown, the products are absent in the chemical databases, and the researcher needs to make many attempts before a plausible structure is proposed. For discovering more and more UDMH transformation products, special software was made that can also be applied to any other similar tasks of the non-target analysis. The software with a graphical user interface allows for drawing the structures of candidates with the embedded molecular editor to predict in one click an electron ionization mass spectrum, retention indices on several stationary phases, and an MS^2^ mass spectrum. For mass spectra prediction, CFM version 2.4 is used [33,34], and for the retention index, the previously published deep learning models [30,36] are used. All predicting models and libraries are embedded into the ready-to-use software. The software computes the following similarity measures (following the previous works [33,37]) between the observed and predicted spectra: the weighted dot product similarity (*S_dp_*), Jaccard similarity (*S_j_*), weighted recall (*S_wr_*), and weighted precision (*S_wp_*):

(1) $$S_{dp}=\frac{{\left(\sum_{n=m}^{M}n^{2}p_{n}^{0.5}t_{n}^{0.5}\right)}^{2}}{\sum_{n=m}^{M}\left(n^{2}p_{n}\right)\sum_{n=m}^{M}\left(n^{2}t_{n}\right)}$$

(2) $$S_{j}=\frac{N_{pt}}{N_{p}+N_{t}-N_{pt}}$$

(3) $$S_{wr}=\frac{I_{t1}}{I_{t}}$$

(4) $$S_{\mathrm{wp}}=\frac{I_{p1}}{I_{p}}$$

where *p_n_*, *t_n_*—intensities of predicted and target (observed) spectra at *m*/*z* = *n*; *m*—starting *m*/*z*; *M*—maximal *m*/*z*; *N_p_*, *N_t_*, *N_pt_*—number of peaks in the predicted spectrum, target spectrum, and number of peaks presented in both spectra simultaneously, respectively; *I_t_*_1_, *I_p_*_1_—sums of intensities of peaks presented in both spectra simultaneously computed for target and predicted spectra, respectively; *I_t_*, *I_p_*—sums of intensities of all peaks for target and predicted spectra, respectively.

**Figure 3:**
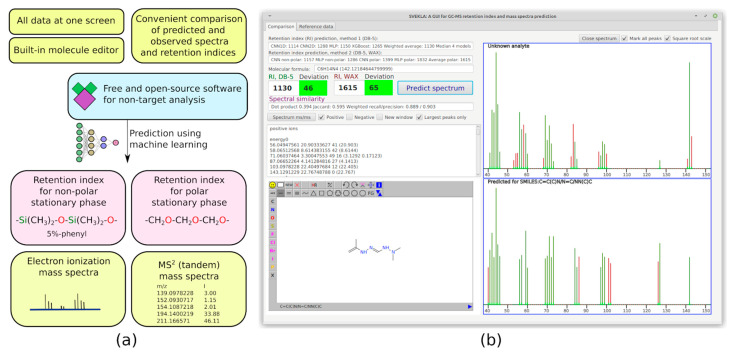
(**a**) Functionality of the developed software; (**b**) Sample screenshot of the software.

Since the selection of candidates based only on matching factors between the predicted and observed spectra is not a reliable approach, the existing software such as CFM-ID cannot be used alone. There is no alternative software that combines the predicting models for spectra of various types and retention, and that allows for manual comparison in a convenient way. We hope that our software can be used in further research devoted to non-target analysis. The overview of the software and an example screenshot are presented in Figure 3. The Java programming language is used for the development of this software. The software is completely offline, free, and open-source, and it can be downloaded from the GitHub repository: https://github.com/mtshn/svekla (accessed on 1 April 2023).

### 2.3. Critical Assessment of the Workflow and the Previously Discovered Compounds

As a model object for analysis, a mixture of the UDMH transformation products was considered. The mixture was formed during the long-term uncontrolled storage of UDMH-containing wash water. The mixture was described in previous works [16,17,29]. This mixture contains dozens and dozens of UDMH transformation products, and the structures of most of them are unknown and absent in any chemical databases [17]. In the previous work [17], the structures of 24 transformation products were proposed by our team. The novel GC-MS and HPLC-HRMS workflow with five criteria was used. The addition of two more criteria: RI on the polar stationary phase and MS^2^ (see Section 2.1) allows us to crosscheck these structures and the evaluation accuracy of the workflow.

**Figure 4:**
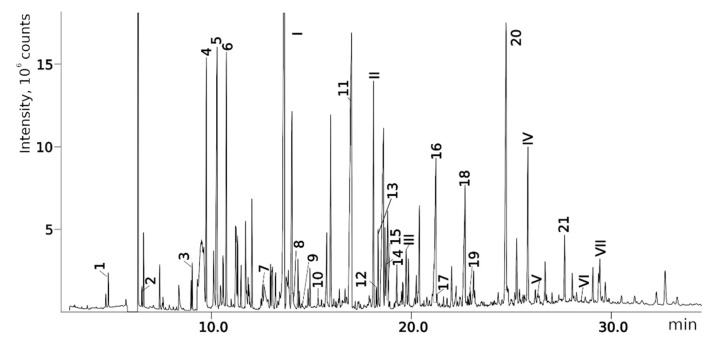
Mass chromatogram (gas chromatography and total ion current) of the considered mixture, registered using the polar stationary phase. Peaks are signed according to the compound numbers listed in Table 2 and Table 3.

Figure 4 shows a chromatogram of the mixture under consideration on the polar stationary phase (see Section 4.1). The examples of the GC-MS mass chromatograms of this mixture on the non-polar stationary phase and the HPLC-MS mass chromatograms of this mixture are presented in previous works by our team [16,17]. Some data are also presented in the Supplementary Materials published alongside these works [16,17]. In Table 2, the results of the re-evaluation of 24 structures proposed in the previous work [17] are shown. In 15 of 24 cases (compounds **1**, **3**, **5**, **6**, **8**, **9**, **10**, **11**, **13**, **15**, **16**, **17**, **18**, **19**, **20**; numbers are listed according to Table 2), the previously established structures passed the crosscheck and were confirmed using two new criteria: MS^2^ and RI on the polar stationary phase. In one case (compound **7**) in the previous work, there was an ambiguity that was solved using the new criteria—only one of two candidates passes the new workflow. In one case (compound **14**), the previously proposed structure satisfies all new criteria, but a new candidate that also satisfies all criteria was proposed. In four cases (**2**, **4**, **12**, **21**), the previously established structures did not satisfy the new criteria, and new structures that fit all new criteria were proposed. In two of four cases, there are other positional isomers with very similar structures: a different position of a heteroatom or a substituent in the aromatic ring. In three cases, the corresponding compounds in the chromatogram measured using the polar stationary phase were not detected. Overall, in 17 of 24 (70%) cases, new criteria were satisfied for the proposed structures. The main conclusion that can be made at this point is the extreme complexity of the structure elucidation task based only on chromatographic and mass spectral data. Multiple structures can even satisfy multiple criteria at once, and any “preliminary identification” that uses only one method and one criterion is very unreliable. Unfortunately, such approaches were often used in previous works on UDMH transformation products (see Table 1). At the same time, the complex approach with several criteria is more reliable, and in the vast majority of cases, it allows for the determination of structure correctly or almost correctly (e.g., up to positional isomerism in the aromatic ring).

### 2.4. The Newly Proposed Structures of UDMH Transformation Products

The newly proposed (compared with the previous work [17]) structures of UDMH transformation products are shown in Table 3. All of these structures meet all the considered criteria.

Five of these structures have never been considered before and are newly proposed, and two of the seven listed structures (compounds **V**–**VI**) are the confirmation of those previously reported [16] (there, these molecules were identified using NMR). These two structures are absent in the work [17], because when using RI only on the non-polar stationary phase, it was not possible to unambiguously match the peaks on the GC-MS mass chromatogram with the structures. Now, we can consider these structures as being confirmed using the updated workflow. Since the structures were previously confirmed using NMR, this fact demonstrates the robustness of the workflow.

The acute toxicity of these compounds was estimated using the OPERA 2.9 software [38,39]. The results are shown in Table 3. To calculate the toxicity, the graphical user interface was used, the “AcuteTox (CATMoS)” endpoint was selected, and the “Standardize” option was turned off. For all compounds except compounds **II** and **III**, the median lethal dose was estimated to be in the range of 50–500 mg/kg (EPA category [39] II). For the two remaining compounds, a median lethal dose is about 900 mg/kg (EPA category [39] III). Since UDMH itself belongs to EPA category II, five of the seven compounds have an acute toxicity that is comparable to UDMH.

## 3. Discussion

Let us consider in more detail the cases where the use of the polar stationary phase and the corresponding RI made it possible to refine the structures of the proposed candidates. The use of the RI for the polar stationary phase makes it possible to solve the ambiguities that remained in our previous work [17]. This is observed with the candidates 1H-pyrazole, 3,5-dimethyl- and 1-methylpyrrol-2-amine (compound **7**). Retention indices on the non-polar stationary phase do not allow for the selection of the best candidate. At the same time, on the polar stationary phase, 1*H*-pyrazole, 3,5-dimethyl- (predicted RI—1678), and 1-methylpyrrol-2-amine (predicted RI—1858) have a large difference in RI. Additionally, if we compare these RIs with the experimental value of 1591, it is unambiguous that 1*H*-pyrazole, 3,5-dimethyl- is a more suitable structure. However, there are examples (compounds **3** and **5**) for when the use of RI for the polar stationary phase still does not allow for the choice of a single candidate. It is not possible to distinguish these isomers using only the mass spectra.

Previously, pyrazole, 1,4-dimethyl- was proposed as one of the candidates for transformation products (compound **2** in Table 2). The difference between the predicted and the experimental RI is 151 units for the polar stationary phase. So, this structure can be rejected. At the same time, pyrazole, 1,3-dimethyl- (the observed and predicted RI are 1215 and 1273 for the polar stationary phase, respectively) is suitable. Both compounds satisfy the previously considered criteria: pyrazole, 1,4-dimethyl- and pyrazole, 1,3-dimethyl- have similar RIs for the non-polar stationary phase (889 and 834, respectively). The same situation occurs with the previously proposed *N*-ethyl-*N*-methyl-1,2,4-triazol-1-amine (compound **4**). It satisfies all of the previously proposed criteria, but the use of the RI for the polar stationary phase results in the rejection of this structure. *N*-ethyl-*N*-methyl-1,2,3-triazol-1-amine passes all filters and it is a suitable candidate. Similar situations were observed two more times: see compounds **12** and **21** (Table 2).

Due to the abundance of situations where RI for the polar stationary phase allows for a resolution of the ambiguity, the question arises: is “RI for the non-polar stationary phase” a required criterion, or is the use of the polar stationary phase enough. Previously proposed [17], *N*5-((dimethylamino)methyl)-*N*4-methyl-1*H*-imidazole-4,5-diamine (compound **21**) does not match the RI for the polar stationary phase. The difference between the experimental and the predicted RI is 516 units. Another possible structure is the following:
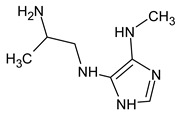
 For this compound, the difference between the indices on the polar stationary phase is 11 units, but such a candidate does not satisfy the condition for the difference in indices of 70 units for the non-polar stationary phase. So, the following structure satisfies all the criteria, including RI for two stationary phases:
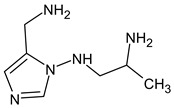
 So, it can be concluded that if RI for the polar stationary phase is used alone, there are also ambiguities, and the use of two RI increases the reliability.

In one case (compound **14**), a new candidate that satisfies all criteria was also proposed. In the previous work [17], if we found a candidate from the NIST mass spectral database that fits all criteria, we did not search for any new candidates. During the course of this work, we found that a new candidate can also be proposed, in addition to the already chosen one. This case demonstrates one more time that even when using many criteria, the structure determination based only on chromatography and mass spectrometry (including retention data and HRMS) is not thoroughly reliable.

Another interesting example includes three isomeric compounds (compounds **18**–**20** in Table 2) with a molecular weight of 153. The proposed structures meet all criteria and were previously confirmed using NMR [16]. However, none of the previously used methods allows for a confirmation of the order of atoms in the substituent (including NMR). For example, the following structure:
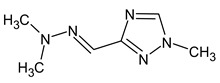
 meets all of the previously used criteria: NMR and GC-MS (with RI for the non-polar stationary phase), and MS^2^. Nevertheless, the use of RI for the polar stationary phase allows for the rejection of such a structure and the confirmation of the only one possible order of atoms:
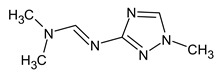


In three cases (compounds **22**–**24**), the previously found [17] compounds were not found on the mass chromatogram registered using the polar stationary phase. In one of the cases (compound **23**), it is obvious that the compound co-elutes with the solvent, and that the mass spectra are not registered for these retention times (the so-called “solvent cut” occurs). Such a hypothesis is consistent with the predicted RI on the polar stationary phase. In two other cases, it is unknown as to why the compounds (both are amides) were not observed. Hence, in this work, we cannot confirm the presence of these molecules in the mixture under analysis.

To illustrate the importance of RI for two stationary phases, two more examples were considered. For example, the following compound: 
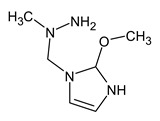
 meets all criteria for compound **III** (Table 3) except for RI for the non-polar stationary phase. Both the compound proposed in Table 3 and this compound meet all other criteria and do not contradict the observed mass spectral data. So, the RI for the polar stationary phase is not enough. The multiple examples of how RI for the polar stationary phase allows for a resolution of the ambiguity, are given above.

Another similar example is compound **II** (Table 3). The structure
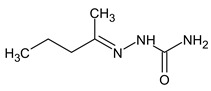
 does not contradict the information on RI for the non-polar stationary phase, but it has a difference of 281 between the predicted and observed RIs for the polar stationary phase. On the contrary, the structure
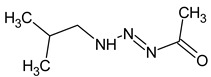
 has an appropriate RI for the polar stationary phase, and a large difference between the predicted and observed RIs for the non-polar stationary phase (and it is also highly likely to be unstable). The structure proposed in Table 3 meets all of the criteria. All of the mentioned structures do not contradict the available mass spectral data.

Finally, it should be demonstrated that RI for two stationary phases (even with a known molecular formula) are absolutely not enough to determine the structure. We have observed one more peak of the unknown compound that most likely has a molecular weight of 223 but which differs from the compounds presented in lines **V**–**VI** of Table 3. The following compounds met the RI criteria (for both stationary phases) for this peak:
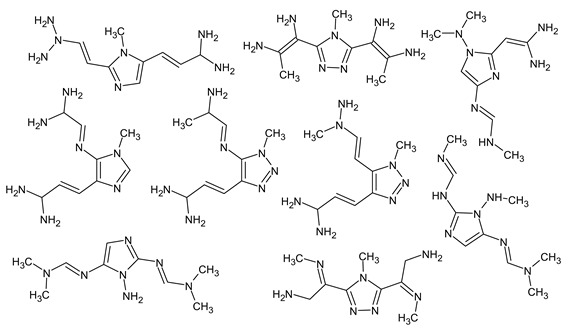
 and there are probably many more similar structures. All of them have the same molecular formula. However, all of them contradict the available mass spectral data, and this peak remains undeciphered.

The developed methodology can be used only with volatile and thermally stable UDMH transformation products, since it relies on GC-MS. If a compound undergoes destruction in an injector of a gas chromatograph, and the destruction product is detected in GC-MS, this product will be rejected by the HPLC-HRMS criteria of our workflow. So, such false positive detections are unlikely. The isomerization in the injector cannot be ruled out completely, but the distinction of isomers is anyway not always reliable. However, we consider such destruction to be relatively unlikely, since the structures of quite heavy (molecular weight 153 and 223) UDMH transformation products confirmed via NMR were also confirmed via GC-MS in unaltered form.

Thermally unstable UDMH transformation products cannot be detected using the considered workflow. The detection of such compounds has to rely only on liquid chromatography. Fortunately, creating such a multimodal approach using only HPLC-MS is also possible. There are quite accurate methods of retention time prediction for liquid chromatography based on deep learning [40,41,42]. Moreover, while RI for only few stationary phases is available in GC-MS, prediction for various stationary phases and eluent compositions is possible in HPLC-MS [42,43]. Furthermore, an additional criterion that can be used is the collision cross-section in ion mobility spectroscopy [44]. The prediction of these values for small molecules and peptides using machine learning has been extensively studied in recent years [45,46,47]. The use of HPLC retention times and collision cross-sections for the rejection of false candidates was demonstrated [41,46,48].

## 4. Materials and Methods

### 4.1. Materials

A blend of n-alkanes C_8_–C_40_ (0.5 mg/mL of each component in dichloromethane, Sigma-Aldrich, St. Louis, MO, USA) was used for the determination of RI. MS-grade acetonitrile (AppliChem, Darmstadt, Germany) was used for sample dilution and HPLC-HRMS. The following chromatographic columns (polar stationary phase and non-polar stationary phase, respectively) were used for GC-MS: SH-Stabilwax capillary column (30 m × 0.25 mm × 0.10 μm, Shimadzu Corporation, Kyoto, Japan) and HP-5 capillary column (30 m × 0.32 mm × 0.25 μm, Agilent, Santa Clara, CA, USA). An InfinityLab Poroshell 120 EC-C18 analytical column (4.6 × 100 mm, particle size 2.7 μm, Agilent, USA) was used for HPLC-HRMS.

A representative sample (0.1 g, dark resinous liquid) was taken from a mixture formed from the long-term storage of UDMH-containing waste [16,17,29]. The sample was diluted in 1 mL of acetonitrile and centrifuged for 20 min. The supernatant was injected into GC-MS (with the polar stationary phase).

### 4.2. GC-MS

The GC-MS parameters and conditions were similar to those used in the previous work [17]. A Shimadzu GCMS-TQ8040 system (Shimadzu Corporation, Kyoto, Japan) was used to perform GC-MS analysis. GC-MS parameters (polar stationary phase) were used as follows: an injection volume of 1 μL, a sample injection temperature of 250 °C, a split injection mode with a ratio of 1:5, and a carrier gas flow rate of 1.0 mL/min. Temperature program: 3 min at 50 °C, ramping the temperature to 250 °C at a rate of 8 °C/min, then 9 min at 250 °C; ion source temperature 200 °C and scanning range 40–500 *m*/*z*. The conditions used for a non-polar stationary phase were the same except for the carrier gas flow rate and temperature program—flow rate: 1.46 mL/min; program: 3 min at 50 °C, then ramping to 320 °C at a rate of 8 °C/min, then 5 min at 320 °C. The NIST 17 (NIST, Gaithersburg, MD, USA) database and the AMDIS software, version 2.3 (NIST, USA) were used for the library search and for deconvolution, respectively. Linear RIs based on n-alkanes were used. More details are provided in the previous work [17].

### 4.3. HPLC-MS^2^

For HPLC-MS^2^ analysis, an Agilent 1260 Infinity chromatographic system (Agilent, USA) and a Bruker Maxis Impact QTOF mass detector (Bruker, Leipzig, Germany) were used. Chromatographic conditions were described in the previous work [17]. The MS detection parameters were as follows: electrospray ionization (ESI), positive ion mode, range 80–500 *m*/*z*, source temperature of 200 °C, drying gas flow: 7 L/min, nebulizer gas pressure: 1.4 bar, and capillary voltage: 4.5 kV. The mass determination error did not exceed 0.03 ppm.

Tandem mass spectrometry was performed in collision-induced dissociation mode for a selected range of molecular ions with an *m*/*z* value of more than 100. Nitrogen was used as the collision gas, and the collision energy was 10 eV. The width of the *m*/*z* range selected with quadrupole for the precursor ion MS^2^ acquisition was ±0.5.

## 5. Conclusions

UDMH, a widely used rocket propellant, forms a variety of oxidative transformation products that can harbor significant health and environmental dangers. The elucidation of the structures of these compounds is an important task since many regions of the Earth have been contaminated. In previous works, either well-known transformation products were determined using target analysis, or very unreliable methods were used in order to propose the structures of transformation products in non-target analysis. In this work, a new workflow was developed that uses a combination of several methods of chromatography and mass spectrometry (seven criteria) for this task. Five new structures of UDMH transformation products were proposed, and four previously proposed structures were refined (see Table 2 and Table 3).

The main conclusion that can be drawn is the absolute necessity to use as many criteria as possible, and to use multiple crosschecks if we propose new UDMH transformation products based only on chromatographic and mass spectrometry data. A shallow analysis of a single observed mass chromatogram, and the use of only one criterion and approach such as a library search will almost exactly result in incorrect structures. This is also true for other non-target analysis tasks. The proposed approach with seven criteria is more reliable, and the proposed structures are more plausible. The previously developed approach with five criteria is also relatively reliable, and in most cases, it results in a structure that is confirmed by the application of new criteria. The second conclusion is the importance of RI for two stationary phases. The consideration of two RI allows for the rejection of many candidates that have appropriate RIs for one of the stationary phases and non-appropriate for another. RI allows for the rejection of many candidates that do not contradict the mass spectral information. Future research directions in non-target analysis may include the automatized generation of candidates using the enumeration of isomers with further filtering, and the use of a larger number of criteria (liquid chromatography retention time, ion mobility collision cross-sections, etc.). The improved methodology can help with approaching an exhaustive elucidation of the set of possible UDMH transformation products.

The use of the entire presented workflow became easy because of the user-friendly software developed. The software allows the researcher to try many structures over a manageable amount of time. This software allowed us to propose several new structures of previously unknown UDMH transformation products. The estimated acute toxicity of some newly discovered products is comparable with the acute toxicity of UDMH itself. Finally, it should be concluded that the diversity of the UDMH transformation products is so great that modern research methods do not even allow one to approach the description of all possible UDMH transformation products. Moreover, this diversity may be fraught with underestimated danger.

## Figures and Tables

**Table 1:** Previous works devoted to determination of structures of new, unknown UDMH transformation products.

Methods Used	Sample	Year	Reference
GC-MS, several standard samples of the most common UDMH oxidation products	Synthetic model mixture	1979	Mach et al. [5]
HPLC-MS, GC-MS, standard samples, for 4 of 12 compounds: preparative isolation and NMR	Contaminated soil	2008	Rodin et al. [10]
GC-MS, low resolution, NIST 05 library search	Contaminated soil	2010	Carlsen et al. [8]
GC-MS, low resolution, library search	Synthetic model mixture	2011	Buryak et al. [15]
GC-MS, low resolution, NIST library search	Contaminated soil	2012	Kenessov et al. [12]
HPLC-MS^2^, low resolution, standard samples	Contaminated soil	2014	Kosyakov et al. [9]
HRMS, electrospray ion source, no chromatographic separation	Synthetic model mixture	2017	Ul’yanovskii et al. [14]
HPLC-HRMS, MS^2^, preparative HPLC, NMR (5 compounds)	UDMH-containing wash water after uncontrolled storage	2019	Milyushkin et al. [16]
HRMS, electrospray ion source, no chromatographic separation	Contaminated soil and synthetic model mixtures	2019	Kosyakov et al. [13]
GC-MS, several standard samples of the most common UDMH oxidation products	Synthetic model mixture	2019	Huang et al. [6]
GC-HRMS, library search in the NIST 17 database (low-resolution GC-MS database)	Contaminated soil	2021	Ul’yanovskii et al. [11]
GC-MS with two ion sources, HPLC-HRMS, machine learning for prediction of mass spectra and retention, NIST 17 database	UDMH-containing wash water after uncontrolled storage	2022	Sholokhova et al. [17]
GC-MS, low resolution, NIST 17 library search, some compounds are detected with quite low confidence	Plant samples contaminated with UDMH	2022	Karnaeva et al. [18]

**Table 2:** Proposed structures of detected transformation products of unsymmetrical dimethylhydrazine.

N	RT, min	MW	Structure	RI	Predicted RI
**1** *	4.75	82	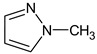	1119	1163
**2** *	6.44	96	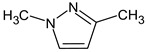 ( 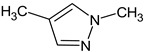 [17])	1215	1273
**3**	9.02	97	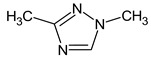 or 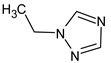	1362	1427 or 1410
**4**	9.72	126	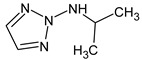 ( 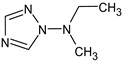 [17])	1406	1500
**5** *	10.24	83	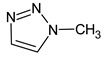 or 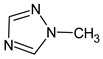	1440	1463 or 1354
**6**	10.71	97	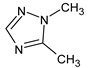	1470	1526
**7** *	12.52	96	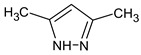 ( 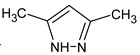 or 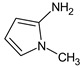 [17])	1591	1680 (NIST)
**8**	14.00	126	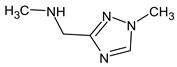	1704	1730
**9** *	14.52	82	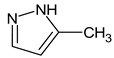	1739	1690 (NIST)
**10** *	15.33	130	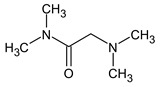	1801	1754
**11** *	16.95	121	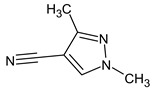	1932	1886
**12**	18.25	152	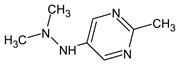 ( 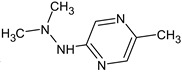 [17])	2041	1963
**13**	18.35	127	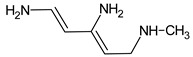	2050	2146
**14** *	18.80	166	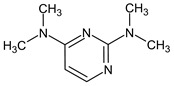 or 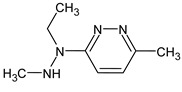 (absents in the NIST 17 database)	2090	2147 or 2079
**15**	18.82	152	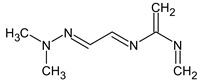	2092	2031
**16**	21.20	112	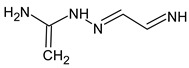	2312	2246
**17**	21.28	153	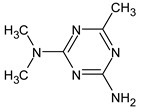	2320	2261
**18**	22.67	153	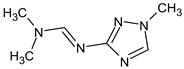	2456	2390
**19**	22.87	153	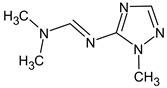	2476	2397
**20**	24.71	153	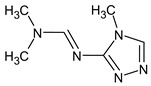	2670	2629
**21**	27.66	169	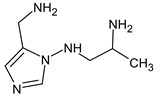 ( 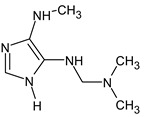 [17])	3005	2927
**22** *	-	102	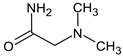	Not detected	
**23** *	“solvent cut”	85	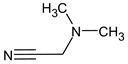	Not detected	
**24** *	-	73	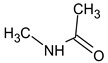	Not detected	

* Asterisks denote compounds for which the NIST 17 database contains reference mass spectra. Retention time (RT) and retention index (RI) for the polar stationary phase are listed. In two specially denoted cases, the reference RI are available in the NIST database, and these values are listed instead of the predicted ones. For such cases when the structures of proposed candidates were refined using new criteria, the previously proposed structures are indicated in parentheses.

**Table 3:** Proposed structures of detected unsymmetrical dimethylhydrazine transformation products. Retention time (RT) and retention index (RI) for the polar stationary phase, and RI for the non-polar stationary phase are given. The EPA acute toxicity category is also given.

			Polar Stationary Phase		Non-Polar Stationary Phase		
N	RT, min	Structure	RI	Predicted RI	RI	Predicted RI	EPA Category
**I**	13.65	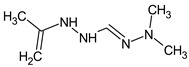 or 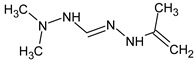	1681	1615	1177	1131	II
**II**	18.11	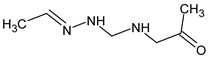	2030	2077	1295	1275	III
**III**	19.74	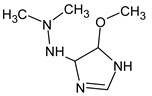	2175	2255	1427	1387	III
**IV**	25.82	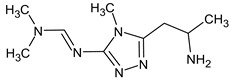	2792	2768	1841	1821	II
**V**	26.34	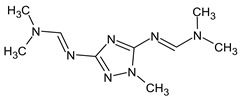	2851	2879	1983	1944	II
**VI**	28.75	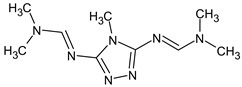	3132	3027	2010	1994	II
**VII**	29.43	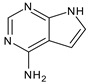 or 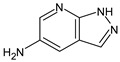	3201	3240	1733	1726	II

## Data Availability

The authors confirm that the data supporting the findings of this study are available within the article. The open-source software developed by the authors is available online: https://github.com/mtshn/svekla (accessed on 1 April 2023).
